# An Empirical Investigation of Transfer Effects for Reinforcement Learning

**DOI:** 10.1155/2020/8873057

**Published:** 2020-12-16

**Authors:** Jung-Sing Jwo, Ching-Sheng Lin, Cheng-Hsiung Lee, Ya-Ching Lo

**Affiliations:** ^1^Master Program of Digital Innovation, Tunghai University, Taichung 40704, Taiwan; ^2^Department of Computer Science, Tunghai University, Taichung 40704, Taiwan

## Abstract

Previous studies have shown that training a reinforcement model for the sorting problem takes very long time, even for small sets of data. To study whether transfer learning could improve the training process of reinforcement learning, we employ Q-learning as the base of the reinforcement learning algorithm, apply the sorting problem as a case study, and assess the performance from two aspects, the time expense and the brain capacity. We compare the total number of training steps between nontransfer and transfer methods to study the efficiencies and evaluate their differences in brain capacity (i.e., the percentage of the updated Q-values in the Q-table). According to our experimental results, the difference in the total number of training steps will become smaller when the size of the numbers to be sorted increases. Our results also show that the brain capacities of transfer and nontransfer reinforcement learning will be similar when they both reach a similar training level.

## 1. Introduction

Reinforcement learning (RL) aims at learning policies to map from states to actions for the purpose of maximizing the expected accumulated reward and reaching the goal. Compared with the supervised learning approaches where the models are trained on the input set and the given output set, the RL agent has to interact with the environment and learn from those experiences through trial and error to yield the optimal behaviour.

Mathematically, RL can be formulated as a Markov decision process (MDP) which is a framework to model decision-making problems [1]. An MDP is represented by the tuple <*S*, *A*, *T*, *R*> where *S* denotes the state space in the environment and *A* is the action set to take in a given state. Function *T* is defined as *P*(*s*′*|s*, *a*) which indicates the probability of the next state *s*′ ∈ *S*  at time step *t* + 1 given the current state *s* ∈ *S* and the action *a*  ∈  *A* taken at time step *t*. Function *R* is a reward scheme used to assign the score for the action performed under the state *s* and is used as a guidance for the agent to produce suitable behaviours. Then, the objective of the RL agent is to learn a policy *π*_*θ*_(*a|s*) which tells the agent what the best action *a*  ∈  *A* to perform is while the environment is in the state *s*  ∈  *S* with the parameter *θ*. In general, there are two main approaches to solving RL problems, model-based and model-free learning. In the model-based approaches, the goal is to learn the model of the environment and obtain the optimal policy relying on the past transitions. On the other hand, model-free approaches learn to directly acquire the optimal policy by the trial-and-error interactions without modelling the underlying environment. Model-based approaches are often sample-efficient, but the requirement of specifying the model of real-world tasks is often restrictive and difficult to satisfy. Therefore, model-free approach is commonly preferred over the model-based approach if it is not hard to sample the trajectories [2, 3]. Q-learning [4] and SARSA [5] are two well-known model-free RL algorithms which fit the optimized policy by learning the action-value (Q-value) function. Note that an action-value function is used to express the expectation of the reward for each state-action pair (*s*, *a*). In recent years, since the development of deep learning methods has gained significant attention and achieved innovations in many fields, it is common to adopt deep learning methods for RL algorithms in order to boost the performance. A combination of the convolutional neural network (CNN) [6] and Q-learning called deep Q-networks (DQN) [7, 8] is proposed to handle large state-action space. DQNs have been shown to reach at or even beyond human-level performance on many games. An alternative double estimator method, double Q-learning [9], is introduced to reduce the overestimations of the action values in the Q-learning algorithm. As double Q-learning was proposed in a tabular setting and DQN algorithm suffers from overestimations, double DQN is used for large-scale function approximation and to reduce the overestimations by combining double Q-learning and DQN [10–12].

RL algorithms usually require large amounts of trial-and-error and many learning iterations to determine an effective policy from very large-scale state-action space, making them very time-consuming. Recently, there has been a strong interest in the development of deep learning models with the ability to transfer experiences across similar tasks. The two representative types of methods are the transfer of trained models and transfer of learned knowledge [13]. The first methods transform the neural network layers from the pretrained model to the target model [14, 15] whereas the second approaches aim at transferring learned knowledge from the trained network to the target network [16, 17]. A Q-learning-based approach has been applied for the sorting problem [18]. However, it takes large number of training steps to finish the training process, even for small sets of data. Since transfer learning has been widely adopted to speed up the training process, this motivates us to devise a transfer scheme and compare it with the nontransfer method in the training performance. In this paper, we conduct a series of experiments using the sorting problem as a case study. We transfer the knowledge learned from the task *n* to the task *n* + 1 where *n* is the size of the numbers to be sorted and continuously use a Q-learning-based method to train the model. The total number of training steps and the size of the brain capacity, which denotes the knowledge in the Q-table, are two metrics to measure the impact of transfer learning techniques.

The rest of this paper is organized as follows.  Section 2 reviews the background and related work of this paper.  Section 3 describes our training strategies and detailed methodology. Experimental setup and results are presented and discussed in Section 4. In Section 5, we discuss conclusions and future work.

## 2. Background and Related Work

In this section, we first give an overview of Q-learning which is the base RL algorithm in this paper. The application of RL in the sorting problem is discussed as well.

Q-learning, a form of model-free method, is one of the most known RL algorithms initially designed for the use of Markov decision processes. It updates the Q-value with the following rule:

(1) $$\begin{array}{c} \text{Q}\left(s_{t},\;a_{t}\right)⟵\text{Q}\left(s_{t},\;a_{t}\right)+\alpha \left(r_{t}+\gamma \underset{a}{\max}\text{Q}\left(s_{t+1},a\right)-\text{Q}\left(s_{t},\;a_{t}\right)\right), \end{array}$$

where Q(*s*_*t*_,  *a*_*t*_) is the action-value function to compute the expected reward of a state-action pair at time step *t*, *α* is the learning rate, *γ* is the discount factor, and *r*_*t*_ is the reward obtained after selecting action *a*_*t*_ given state *s*_*t*_. The max operator from the update rule indicates that the agent chooses the best action *a* by computing the maximum Q-value for the next state *s*_*t*+1_. The mechanism to exploit the maximum Q-value while updating is called an off-policy algorithm, i.e., the choice of taking action *a*_*t*_ and *a* does not follow the same policy. On the contrary, the SARSA updates the Q-value based on the policy being followed by the following equation:

(2) $$\begin{array}{c} \text{Q}\left(s_{t},\;a_{t}\right)\;⟵\text{ Q}\left(s_{t},\;a_{t}\right)+\alpha \left(r_{t}+\gamma \times \text{Q}\left(s_{t+1},a\right)-\text{Q}\left(s_{t},\;a_{t}\right)\right). \end{array}$$

When the algorithm uses the same mechanism for the behaviour policy (i.e., Q(*s*_*t*_,  *a*_*t*_)) and the estimation policy (i.e., Q(*s*_*t*+1_, *a*)), it is called on-policy [19].

The sorting problem is a quintessential computer science task and has been applied to many fields since its emergence. Based on the analysis of all comparison-based sorting algorithms, the computation requires *O* (*n* log *n*) complexity. A RL-based approach, which applies stability and resiliency ideas from feedback controls, is proposed to overcome the errors and early program termination limitations for the traditional computing [20]. An empirical exploration compares the RL model with two traditional sorting algorithms and shows that the RL sorting model completes the task with less array manipulations. In order to investigate the effect of two different reward schemes, immediate reward and pure delayed reward, a Q-learning algorithm is implemented to compare the total number of training steps and average number of sorting steps [18]. A case study of the sorting problem is conducted and concludes that immediate reward takes much less steps to finish the task.

## 3. Methodology and Learning Design

In this section, we describe important features in our proposed methodology, which include training level and brain capacity. We also discuss how we designed the RL algorithm in order to formulate the sorting problem into RL settings.

### 3.1. RL-Based Setting for Sorting Problem

We model the initial state *s*_0_ Step *t*, which consists of *n* elements, as the list of numbers to be sorted, and hence, there will be *n* factorial possible states denoted by *S*_*n*_. For any state *s* at time, an action *a* (*i*, *j*) is defined as the swap of values in position *i* and position *j*. Thus, there will be $C\left(\begin{array}{c} n\\ 2 \end{array}\right)$ possible actions in the action set *A*_*n*_. Once the action *a*(*i*,  *j*) is chosen under state *s*_*t*_, the next state *s*_*t*+1_ is determined by exchanging the element in position *i* with the element in position *j* of the state *s*_*t*_. For example, assuming the initial state is *s*_0_=[4,  5,  3,  2,  1] and an action *a*(1,  4) is performed, this state-action pair will result in the next state *s*_1_=[2,  5,  3,  4,  1].

As suggested by the previous study [18] that immediate reward performs better than pure delayed reward, we use immediate reward scheme in this research. We give the reward by considering whether the action actually improves the number of elements in the correct position. A similarity value is introduced to measure the similarity between the current state *s*_*t*_ and the goal state *S*_goal_ (i.e., the sorted list) as follows:

(3) $$\begin{array}{c} \text{sim}\left(s_{t},S_{\text{goal}}\right)=\sum_{i=1}^{n}\text{Equal}\left(s_{t}\left[i\right],S_{\text{goal}}\left[i\right]\right), \end{array}$$

where Equal function will return one if two states have the same value at position *i* and zero otherwise. We then compute the difference of sim(*s*_*t*_, *S*_goal_) and sim(*s*_*t*+1_, *S*_goal_) to assign the reward as follows:

(4) $$\begin{array}{c} \text{Reward}=\left\{\begin{array}{cc} \text{reward\_better,} & \text{sim}\left(\;\right.s_{t+1},\;S_{\text{goal}}\left.\;\right)>\text{sim}\left(\;\right.s_{t},\;S_{\text{goal}}\left.\;\right),\\ \text{reward\_equal,} & \text{sim}\left(\;\right.s_{t+1},\;S_{\text{goal}}\left.\;\right)=\text{sim}\left(\;\right.s_{t},\;S_{\text{goal}}\left.\;\right),\\ \text{reward\_worse, } & \text{sim}\left(\;\right.s_{t+1},\;S_{\text{goal}}\left.\;\right)<\text{sim}\left(\;\right.s_{t},\;S_{\text{goal}}\left.\;\right). \end{array}\right. \end{array}$$

In this paper, reward_better is 1, reward_equal is 0, and reward_worse is −1. For the aforementioned example, since *s*_0_=[4,  5,  3,  2,  1] will receive a similarity value of 1 and *s*_1_=[2,  5,  3,  4,  1] will receive a value of 2, the reward value of reward_better will be given.

### 3.2. Learning Algorithm

The objective of the learning algorithm is to sort a given example which consists of *n* numbers for a series of episodes until the success rate reaches a predefined threshold.  Algorithm 1 (RL_Sort) represents how we executed the model training on one training instance based on the Q-learning algorithm. The algorithm gives a list *S*_training_ and a Q-table as inputs and then produces a new Q-table and the number of training steps as output. RL_Sort begins with the initialization of upper_bound, train_steps, and success_rate. The upper_bound is used to define the maximum allowed number of swaps for sorting and we set *n* + 1 as the threshold. The variable train_steps is to store the number of episodes spent for training. The variable success_rate is the criterion to terminate the training process and is set to 0.75 in our experiments. *S*_goal_ is the correct sorting result. The experimental parameters are as follows: *α* = 0.05, *γ* = 0.9, and *ε* = 0.85. In each episode from line 11 to line 31, the model chooses an action *a*(*i*, *j*) given current state *s* based on *ε*-greedy [21] and receives a new state s' (lines 12∼13). There are two conditions in which the episode will end. In one condition, *s*′ is the *S*_goal_ and a positive reward (reward_win = 1) will be given (lines 16∼18). In the other condition, the number of swapping times already exceeds upper_bound and a negative reward (reward_lose = −1) will be received. Since the first condition reaches a success state, we will examine the success rate for the latest 100 episodes to determine whether the training process should stop or a new episode should begin. For the cases that the current episode needs to continue (lines 23∼28), the Q-table is updated based on the reward equation (4).

**Figure 1:**
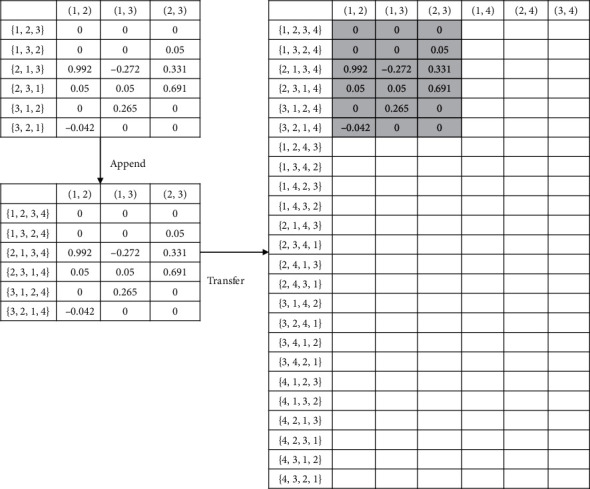
An illustration of transferring the Q-table from *n* = 3 to *n* = 4.

When the training task moves from the example of sorting *n* numbers to *n* + 1 numbers, values in Q-table are usually set to zero or randomly initialized. In our transfer setting, the knowledge learned from sorting *n* numbers is migrated to solve the problem of sorting *n* + 1 numbers. For the Q-table obtained from sorting *n* numbers (denoted as Q_source with size $\left(n!\right)\times C\left(\begin{array}{c} n\\ 2 \end{array}\right)$), we expand its state representation by appending a number *n* + 1 at the end of each state to fit in the Q-table representation for sorting *n* + 1 numbers (denoted as Q_target with size $\left(n+1\right)!\times C\left(\begin{array}{c} n+1\\ 2 \end{array}\right)$). Therefore, each state *s* in Q_source will become s.append(*n* + 1). We then are able to map the Q-value of the state-action pair from Q_souce to Q_target. In this way, as the number in position *n* + 1 is already in the correct position, we try to encourage the model to exploit the prior knowledge from Q_souce and avoid touching the action related to the position *n* + 1. For example, when *n* equals 3 and one of the state is [1, 3, 2] with actions *a*(1,  2), *a*(1,  3), and *a*(2,  3), we will transfer these 3 Q-values in Q_source to Q_target where the corresponding state is [1, 3, 2, 4] with actions *a*(1,  2), *a*(1,  3), and *a*(2,  3). Those nontransferable Q-values will be set to zero or randomly initialized.  Figure 1 demonstrates how we transfer a Q-table from *n* = 3 to *n* = 4.

### 3.3. Performance Metrics

In this paper, we define three performance metrics which include training level, number of training steps, and brain capacity.

*Training level* is a performance-oriented indicator to measure how well the model can use the existing knowledge to perform the task during training. After finishing a training procedure of one instance for sorting *n* numbers, the model is scheduled to sort *n*! tasks where each task is given by a permutation of those *n* numbers. Subsequently, we compute the average number of sorting steps for these *n*! tasks as the model's training level. *Number of training steps*, which is denoted as train_steps in Algorithm 1, is the number of episodes that the model spends on training an example. It is an important factor to measure the effectiveness of the algorithm. *Brain capacity* is concerned with the status of Q-table and is an important measure to compare the knowledge usage between nontransfer and transfer methods. It is defined as the ratio of entries which have been updated in a Q-table.

### 3.4. Experimental Setup and Results

In order to compare the difference and efficacy between nontransfer and transfer methods, a case study in the sorting problem is presented. We illustrate a series of experiments for both nontransfer and transfer RL to investigate the difference of training speed and the contrast of knowledge requirement.

#### 3.4.1. Experimental Setup

We design an experimental setting to train the model to sort lists of *n* numbers where each list is from a permutation of {1, 2, ..., *n*}. In order to provide an equitable comparison, we run nontransfer and transfer RL in parallel and propose an algorithm, which is presented as pseudocode in Algorithm 2, to satisfy our needs.

The input of Algorithm 2 consists of a list *S*_training_ which is a permutation of {1, 2, ..., *n*} and a Q-table (TRQ_*n*−1_[*S*_*n*−1_, *A*_*n*−1_]) which is learned from sorting *n* − 1 numbers. A Q-table (NRQ_*n*_[*S*_*n*_, *A*_*n*_]) of nontransfer RL is initialized to zero for all Q-values and a Q-table (TRQ_*n*_[*S*_*n*_, *A*_*n*_]) of transfer RL is transferred from TRQ_*n*−1_[*S*_*n*−1_, *A*_*n*−1_] as the mechanism discussed in Section 3. B. A variable upper_bound is used as one of the constraints for the training level. The input list *S*_training_ is given to both *S*_nt_ and *S*_tr_ as the initial sorting list for both methods. Then, the algorithm starts iteratively to solve the sorting tasks. We will begin with the nontransfer RL. This process consists of training and evaluation. In the training part, we input the current Q-tables (NRQ_*n*_[*S*_*n*_, *A*_*n*_]) and the list *S*_nt_ to Algorithm 1 to train the model (line 11). The number of training steps returned from Algorithm 1 is accumulated to the variable NonTrans_Tr_Steps (line 13). For the evaluation part, the returned NRQ_*n*_[*S*_*n*_, *A*_*n*_] of Algorithm 1 is then used to sort *n*! lists from the permutation of {1, 2, ..., *n*} and the average number of sorting steps is model's training level denoted as Avg_nt_. We then select the list which takes the maximum number of steps to sort as the new *S*_nt_ (line 15). The same procedure is also applied for transfer RL as seen in lines 12, 14, and 16. The above process is repeated until two models reach a similar training level (i.e., Avg_nt_ and Avg_tr_ are very close or both of them are lower than upper_bound). This restriction is to ensure that both two methods exhibit comparable abilities to sort *n*! lists and affirm that it is fair to conduct a further comparison of the total number of training steps and the brain capacity.

#### 3.4.2. Experimental Results

As an empirical study, we illustrate our results for *n* equal to 5, 6, 7, and 8. In order to produce a more fair view of the comparison, we repeat Algorithm 2 for 30 episodes for each *n*. The total number of training steps and the brain capacity are two perspectives to measure the performance. The total number of training steps for the nontransfer method is abbreviated to NonTrans_Tr_Steps and Trans_Tr_Steps for the transfer method. These two variable names are used in Algorithm 2 as well. Subsequently, we apply similar abbreviations to the brain capacity and denote them by NonTrans_Br_Capacity and Trans_Br_Capacity. NonTrans_Br_Capacity is calculated as the ratio of Q-values which have been updated in the NRQ_*n*_[*A*_*n*_, *S*_*n*_] and Trans_Br_Capacity is the percentage of updated Q-values in the TRQ_*n*_[*S*_*n*_, *A*_*n*_]. The detailed results are reported in Tables 1–4 for different *n*. Looking at the comparison of the total number of training steps, we can see that the values of NonTrans_Tr_Steps and Trans_Tr_Steps increase significantly when *n* increases. It is worth noting that some of these two values are less than 100 when *n* is 5. Therefore, instead of using the latest 100 episodes to check the success rate mentioned in Section 3. B, we opt for the latest 10 episodes to examine that. Regarding the comparison of the brain capacity, the values of NonTrans_Br_Capacity and Trans_Br_Capacity are generally smaller than 0.25 and their values are almost less than 0.1 while *n* is greater than 6. This implies that the knowledge requirement only occupies a small portion of the Q-table in order to solve the sorting task.

For each episode, we also calculate the ratio of the total number of training steps (Ratio_Tr_Steps) as the division of NonTrans_Tr_Steps by Trans_Tr_Steps and the ratio of the brain capacity (Ratio_Br_Capacity) as the division of NonTrans_Br_Capacity by Trans_Br_Capacity. For the value of Ratio_Tr_Steps, there are nine numbers greater than or equal to 5.00 when *n* equals 5. But, as *n* increases, this phenomenon does not appear and the transfer effects diminish. For the value of Ratio_Br_Capacity, the range is much narrower and is largely concentrated between 0.75 and 1.25. As described in Algorithm 2, both nontransfer and transfer methods are required to have very close training levels in order to finish a training episode. Since close training level means that two methods have similar abilities and performance for sorting *n*! lists, this could explain why the value of Ratio_Br_Capacity is around 1. In general, transfer method exhibits better performance in terms of training steps. However, in some cases, Ratio_Tr_Steps is smaller than 1, which means nontransfer method takes less steps to complete the training. Since both methods require similar size of the brain capacity to sort *n*! lists, there may be possibilities that the transfer model exploits the transferred knowledge but does not explore enough to expand its knowledge. This will lead to take more training steps to finish the training process.

**Figure 2:**
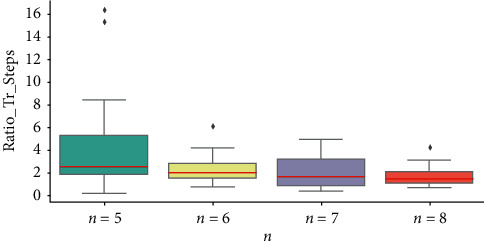
The boxplots of Ratio_Tr_Steps for different *n*.

**Figure 3:**
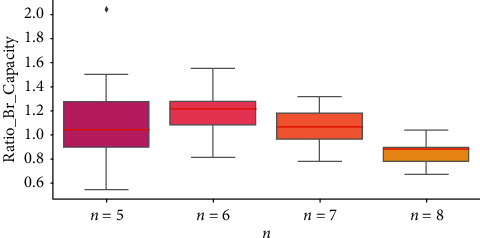
The boxplots of Ratio_Br_Capacity for different *n*.

To explore the distribution of the Ratio_Tr_Steps and Ratio_Br_Capacity, boxplots are presented in Figures 2 and 3 to do the statistical analyses. A boxplot represents the minimum, 25^th^ percentiles, median, 75^th^ percentiles, and maximum of the given dataset. In Figure 2, we observe that the medians of the Ratio_Tr_Steps, which are the red lines inside the box, gradually decrease when *n* increases. This is in accordance with our previous observation that the growth of *n* may lower the transfer effects. In Figure 3, the medians of the Ratio_Br_Capacity all occur around 1.00 mostly aligning with our previous conjecture. In addition to the statistics in boxplots, we also compute the averages of Ratio_Tr_Steps and Ratio_Br_Capacity in Table 5. The average performance shows very similar trends as the boxplots.

## 4. Conclusions

It is reported from prior research that the Q-learning-based approach for the sorting problem requires a large number of training steps. Since the transfer learning method is able to share the knowledge learned from the source domains with the target domain, we devised a transfer scheme to investigate the time cost and knowledge usage issues between nontransfer and transfer models. The Q-table obtained from the prior task is served as the knowledge source to be transferred to the next task. We chose the sorting problem as our case study to analyse two important performance metrics, number of training steps and brain capacity. As a result of the experiment, the brain capacity for two models will be similar after reaching a similar training level. The difference of the total number of training steps between two models will be significant when *n* is smaller. However, as *n* increases, the proportion of the transferred knowledge will be smaller and the difference will become less pronounced, making the transfer effect insignificant.

As shown in Table 4, the maximum number of total training steps is close to 100,000 while *n* equals 8. It would be necessary to enable faster learning in order to handle larger *n*. Future work will therefore be concerned with the reduction of the state space. State abstraction [22, 23] with the ability to leverage the knowledge learned from prior experiences is worth the effort to improve the scalability of the current approach. Another area of future work is to extend the current tabular representation approach to the deep learning-based methods in order to improve the learning stability and computational efficiency.

## Figures and Tables

**Algorithm 1:**
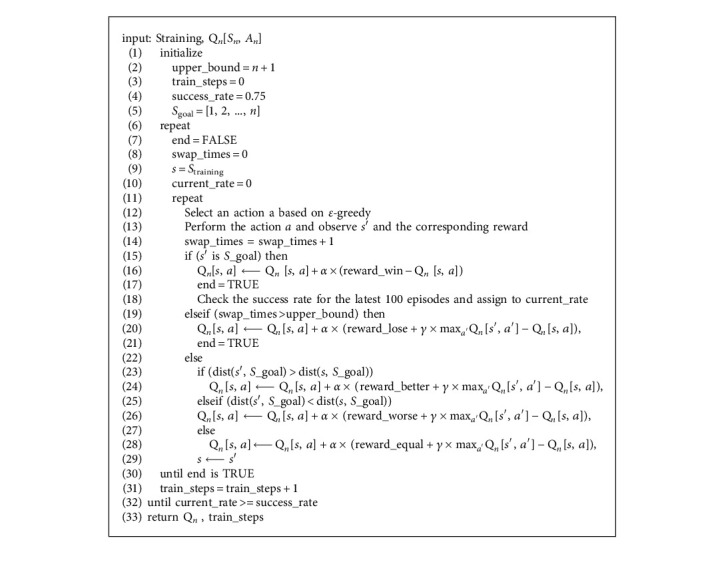
The Q-learning based algorithm for the sorting task. RL_Sort.

**Algorithm 2:**
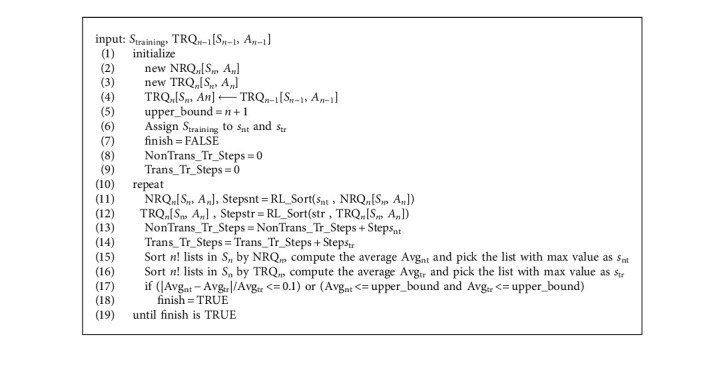
The algorithm for training the non-transfer and transfer RL methods.

**Table 1:** Detailed training results of nontransfer and transfer methods to solve sorting 5 numbers for 30 episodes.

*n* = 5						
	NonTrans_Tr_Steps	Trans_Tr_Steps	Ratio_Tr_Steps	NonTrans_Br_Capacity	Trans_Br_Capacity	Ratio_Br_Capacity
0	167	20	8.35	0.1983	0.1500	1.32
1	215	94	2.29	0.1808	0.2342	0.77
2	964	365	2.64	0.2808	0.2142	1.31
3	207	42	4.93	0.2025	0.1533	1.32
4	94	120	0.78	0.1817	0.1717	1.06
5	189	110	1.72	0.1633	0.1825	0.89
6	361	22	16.41	0.2092	0.1783	1.17
7	146	22	6.64	0.1675	0.1642	1.02
8	94	335	0.28	0.1892	0.2083	0.91
9	382	118	3.24	0.2208	0.1742	1.27
10	230	118	1.95	0.1817	0.1850	0.98
11	78	32	2.44	0.1225	0.1775	0.69
12	276	48	5.75	0.1825	0.1517	1.20
13	130	60	2.17	0.2067	0.1525	1.36
14	320	64	5.00	0.2242	0.1658	1.35
15	241	96	2.51	0.1875	0.1883	1.00
16	286	38	7.53	0.2075	0.1633	1.27
17	246	128	1.92	0.2058	0.2042	1.01
18	140	114	1.23	0.1867	0.1567	1.19
19	249	46	5.41	0.3217	0.1575	2.04
20	130	532	0.24	0.1775	0.2183	0.81
21	154	10	15.40	0.0983	0.1000	0.98
22	10	36	0.28	0.0558	0.1042	0.54
23	456	175	2.61	0.2242	0.1842	1.22
24	101	12	8.42	0.0717	0.1175	0.61
25	400	84	4.76	0.1775	0.1183	1.50
26	117	109	1.07	0.0983	0.1283	0.77
27	241	113	2.13	0.1433	0.1633	0.88
28	184	50	3.68	0.2008	0.1858	1.08
29	102	56	1.82	0.1583	0.1617	0.98

**Table 2:** Detailed training results of nontransfer and transfer methods to solve sorting 6 numbers for 30 episodes.

*n* = 6						
	NonTrans_Tr_Steps	Trans_Tr_Steps	Ratio_Tr_Steps	NonTrans_Br_Capacity	Trans_Br_Capacity	Ratio_Br_Capacity
0	936	417	2.24	0.0598	0.0603	0.99
1	1020	508	2.01	0.0878	0.0719	1.22
2	1203	684	1.76	0.1020	0.0725	1.41
3	1253	411	3.05	0.0801	0.0647	1.24
4	750	241	3.11	0.0620	0.0456	1.36
5	1446	1344	1.08	0.1035	0.1137	0.91
6	871	142	6.13	0.0612	0.0395	1.55
7	1386	476	2.91	0.0878	0.0650	1.35
8	972	565	1.72	0.0708	0.0717	0.99
9	1272	752	1.69	0.0874	0.0746	1.17
10	857	426	2.01	0.0697	0.0560	1.24
11	1175	3850	0.31	0.1052	0.1300	0.81
12	1199	563	2.13	0.0882	0.0673	1.31
13	945	543	1.74	0.0809	0.0710	1.14
14	1634	915	1.79	0.1110	0.0873	1.27
15	1281	944	1.36	0.0971	0.0956	1.02
16	970	628	1.54	0.0847	0.0780	1.09
17	1070	428	2.50	0.0719	0.0593	1.21
18	3918	4929	0.79	0.1569	0.1664	0.94
19	1578	955	1.65	0.1133	0.0908	1.25
20	857	203	4.22	0.0530	0.0437	1.21
21	1461	1008	1.45	0.1050	0.0975	1.08
22	743	364	2.04	0.0639	0.0534	1.20
23	1299	633	2.05	0.0866	0.0734	1.18
24	1665	686	2.43	0.1037	0.0734	1.41
25	4582	1216	3.77	0.1469	0.1098	1.34
26	945	695	1.36	0.0768	0.0680	1.13
27	4021	1201	3.35	0.1384	0.1086	1.27
28	942	474	1.99	0.0737	0.0597	1.23
29	1453	1276	1.14	0.1109	0.1165	0.95

**Table 3:** Detailed training results of nontransfer and transfer methods to solve sorting 7 numbers for 30 episodes.

*n* = 7						
	NonTrans_Tr_Steps	Trans_Tr_Steps	Ratio_Tr_Steps	NonTrans_Br_Capacity	Trans_Br_Capacity	Ratio_Br_Capacity
0	7444	3725	2.00	0.0575	0.0476	1.21
1	17430	10013	1.74	0.0895	0.0761	1.18
2	10969	3716	2.95	0.0605	0.0494	1.22
3	11175	2908	3.84	0.0541	0.0420	1.29
4	9032	2744	3.29	0.0514	0.0417	1.23
5	3097	731	4.24	0.0257	0.0233	1.10
6	16747	15702	1.07	0.0830	0.0868	0.96
7	6947	4555	1.53	0.0566	0.0524	1.08
8	5964	3726	1.60	0.0502	0.0488	1.03
9	4137	1214	3.41	0.0290	0.0273	1.06
10	11132	12738	0.87	0.0710	0.0782	0.91
11	6983	9039	0.77	0.0594	0.0660	0.90
12	7727	1751	4.41	0.0408	0.0316	1.29
13	12421	28476	0.44	0.0877	0.1083	0.81
14	14832	25429	0.58	0.0920	0.1187	0.77
15	12450	7392	1.68	0.0769	0.0689	1.12
16	10787	6533	1.65	0.0539	0.0529	1.02
17	8659	22808	0.38	0.1045	0.1001	1.04
18	7670	2634	2.91	0.0428	0.0387	1.10
19	8086	9071	0.89	0.0615	0.0659	0.93
20	9687	6631	1.46	0.0556	0.0553	1.00
21	2474	580	4.27	0.0314	0.0296	1.06
22	10906	15964	0.68	0.0664	0.0851	0.78
23	11889	5882	2.02	0.0587	0.0514	1.14
24	5962	4259	1.40	0.0478	0.0493	0.97
25	19346	13054	1.48	0.0886	0.0767	1.16
26	5705	3114	1.83	0.0468	0.0396	1.18
27	7431	12660	0.59	0.0642	0.0762	0.84
28	3096	927	3.34	0.0249	0.0246	1.01
29	4668	953	4.90	0.0337	0.0257	1.31

**Table 4:** Detailed training results of nontransfer and transfer methods to solve sorting 8 numbers for 30 episodes.

*n* = 8						
	NonTrans_Tr_Steps	Trans_Tr_Steps	Ratio_Tr_Steps	NonTrans_Br_Capacity	Trans_Br_Capacity	Ratio_Br_Capacity
0	82101	92246	0.89	0.0624	0.0710	0.88
1	77386	83553	0.93	0.0606	0.0689	0.88
2	32674	17731	1.84	0.0358	0.0452	0.79
3	19818	17490	1.13	0.0340	0.0451	0.75
4	24449	11835	2.07	0.0336	0.0443	0.76
5	34761	27067	1.28	0.0399	0.0487	0.82
6	30299	12635	2.40	0.0348	0.0448	0.78
7	26920	14774	1.82	0.0351	0.0448	0.78
8	53885	44233	1.22	0.0479	0.0546	0.88
9	21150	6778	3.12	0.0293	0.0439	0.67
10	56551	73505	0.77	0.0533	0.0650	0.82
11	47152	43085	1.09	0.0477	0.0544	0.88
12	57590	51508	1.12	0.0505	0.0569	0.89
13	21072	9521	2.21	0.0332	0.0457	0.73
14	57659	36219	1.59	0.0445	0.0500	0.89
15	64347	41492	1.55	0.0523	0.0546	0.96
16	31041	15146	2.05	0.0356	0.0443	0.80
17	72028	72386	1.00	0.0594	0.0678	0.88
18	42305	9869	4.29	0.0393	0.0441	0.89
19	29735	20833	1.43	0.0353	0.0468	0.75
20	50376	46719	1.08	0.0485	0.0559	0.87
21	29481	11004	2.68	0.0452	0.0439	1.03
22	37180	32229	1.15	0.0415	0.0500	0.83
23	42596	25663	1.66	0.0400	0.0473	0.85
24	30466	14245	2.14	0.0337	0.0456	0.74
25	62672	60769	1.03	0.0515	0.0593	0.87
26	57160	55132	1.04	0.0520	0.0538	0.97
27	27488	12747	2.16	0.0466	0.0447	1.04
28	28282	19243	1.47	0.0375	0.0410	0.91
29	34866	26506	1.32	0.0435	0.0477	0.91

**Table 5:** The averages of Ratio_Tr_Steps and Ratio_Br_Capacity for different *n*.

Average item	*n* = 5	*n* = 6	*n* = 7	*n* = 8
Ratio_Tr_Steps	4.12	2.26	2.07	1.65
Ratio_Br_Capacity	1.08	1.18	1.06	0.85

## Data Availability

No data were used to support the findings of the study.
